# Clinicopathological and ultrasound features of endometrial cancer in postmenopausal women: a retrospective study in a single institute in South Korea

**DOI:** 10.11604/pamj.2021.38.148.28101

**Published:** 2021-02-10

**Authors:** Hyen Chul Jo, Jong Chul Baek, Seon Mi Lee, Ji Eun Park, In Ae Cho, Joo Hyun Sung

**Affiliations:** 1 Department of Obstetrics and Gynecology, Gyeongsang National University Changwon Hospital, Gyeongsang National University College of Medicine, Changwon, Korea,; 2 Department of Obstetrics and Gynecology, Gyeongsang National University Hospital, Gyeongsang National University College of Medicine, Jinju, Korea,; 3 Department of Occupational and Environmental Medicine, Gyeongsang National University Changwon Hospital, Gyeongsang National University College of Medicine, Changwon, Korea

**Keywords:** Endometrial cancer, endometrial thickness, endometrial fluid collection, postmenopausal women, transvaginal ultrasound

## Abstract

**Introduction:**

endometrial cancer is the most common type of cancer in the female genital tract. Most patients are diagnosed during postmenopausal periods. This study aimed to investigate the demographic characteristics as well as cutoff value of endometrial thickness and ultrasound characteristics of endometrial cancer in postmenopausal patients.

**Methods:**

we retrospectively analyzed 244 postmenopausal women who underwent endometrial sampling from February 2016 to December 2019. Information of patients was obtained through medical records. The patients were divided into two groups according to histopathological results. Group A included patients with endometrial cancer and group B included patients with non-malignant lesions. Data were summarized based on demographic and ultrasound characteristics.

**Results:**

hypertension and history of endometrial hyperplasia were associated with the incidence of endometrial cancer in this study. Endometrial cancer was diagnosed in all ranges when the endometrial thickness was ≥5 mm. Endometrial fluid collection, with increased endometrial thickness, was a risk factor associated with endometrial cancer.

**Conclusion:**

regardless of symptoms and risk factors, endometrial histological confirmation in postmenopausal women should be conducted immediately if endometrial abnormalities such as an endometrial thickness ≥5 mm or endometrial fluid collection are detected by transvaginal ultrasound.

## Introduction

Endometrial cancer is the most common type of cancer in the female genital tract. It accounts for 1-2% of the total number of cancer-related deaths in Western Europe [1]. Most patients are diagnosed during menopausal periods. The mean age at the time of diagnosis is approximately 63 years [2]. Endometrial cancer is usually classified as type I and type II. Type I represents more than 70% of endometrial cancers and is usually related to unopposed exposure of the endometrium to estrogen. Endometrioid is the most common histological type [3]. It has a good prognosis because of its low grade. These tumors may be associated with microsatellite instability and mutations in phosphatase and tensin homolog, phosphatidylinositol-4,5-bisphosphate 3-kinase catalytic subunit alpha, Kirsten rat sarcoma and Catenin Beta 1 (PTEN, PIK3CA, K-ras and CTNNBI) [4]. Type II accounts for approximately 10% of endometrial cancers, involving serous or clear cell histological types. Type II cases have a poor prognosis because of the high grade, high risk of relapse and metastasis [2,3].

Approximately 10-30% of these tumors are associated with p53 mutations [4]. Endometrial cancer associated with genetic diseases represents up to 10% of the cases and is related to Lynch syndrome (hereditary nonpolyposis colorectal cancer) [5]. Risk factors for endometrial cancer are related to unopposed exposure of the endometrium to estrogen - for instance, early menarche or later menopause or due to tamoxifen use, nulliparity, unopposed estrogen therapy or polycystic ovarian syndrome. Other risk factors that are not related to unopposed estrogen include an age >50 years and metabolic syndromes such as obesity, diabetes mellitus, thyroid disease and Lynch syndrome [2,6-8]. The most common symptom associated with endometrial cancer in postmenopausal women is vaginal bleeding. Approximately 10-20% of postmenopausal vaginal bleeding are diagnosed with endometrial cancer by histological examination [9].

The endometrial thickness (ET) measured by transvaginal ultrasound (TVUS), with the advantages of noninvasiveness, cost-effectiveness and high sensitivity, is used as the initial diagnostic method of choice. However, there is controversy regarding the optimal cutoff value of ET used to determine the need for further invasive procedures for endometrial histological confirmation. Studies have suggested that an ET ≤5 mm should be used as the normal value [10], whereas other investigators have recommended an ET <4 mm or an ET <3 mm [9,11]. Endometrial sampling is recommended for the initial studies to evaluate endometrial cancer along with TVUS. Although dilatation and curettage are preferred, an endometrial sampling using a pipelle is often used to obtain an endometrial tissue sample [12]. In this study, we investigated the demographic characteristics as well as cutoff value of endometrial thickness and ultrasound characteristics of endometrial cancer in postmenopausal patients.

## Methods

Among postmenopausal patients who visited Department of Obstetrics and Gynecology of Gyeongsang National University Changwon Hospital from February 2016 to December 2019, we retrospectively analyzed 244 postmenopausal patients who underwent endometrial sampling during this period, whose both ET measured by TVUS and histologic results were known through medical records. The patients were divided into two groups according to histopathological results. Group A included patients with endometrial cancer and group B included patients with non-malignant lesions (endometrial polyp, submucosal leiomyoma, endometrial hyperplasia, atrophic endometrium, endometritis and insufficient tissue). Menopause was defined as amenorrhea that lasted for at least 12 months in patients >50 years of age. In the case of patients under 50 years of age, follicle-stimulating hormone (FSH) was 30 or more. Endometrial sampling was conducted using a pipelle device or by dilatation and curettage to obtain the samples for histological examinations. In patients undergoing hysterectomy, histological results were obtained those of histological examination were collected after surgery.

Data were summarized according to demographic and ultrasound characteristics. Demographic data were classified into the following characteristics: age, parity, weight, body mass index (BMI), duration of menopause, age of menopause, underlying conditions, menopause hormonal therapy (MHT), tamoxifen use, previous history of diagnosis with endometrial hyperplasia, surgical history and the main complaint at the first time of visiting the clinics. Ultrasound data included the ET, polypoid mass-like lesions and fluid collection within the endometrial cavity. The ET was recorded as the thickest endometrial portion in the sagittal uterine plane using a general ultrasound guideline. Endometrial fluid collection (EFC) involved the anechoic area present within the endometrial cavity. Polypoid mass-like lesions were defined as focal thickening of the endometrial layer based on TVUS examination. Nominal variables were expressed as frequency (n) and percentage (%) and continuous variables were expressed as means (M) with standard deviations (SD). Continuous variables were compared using the student´s t-test. Nominal variables were compared with the use of Chi-squared tests in the case of a theoretical Chi-squared distribution and Fisher´s exact tests in the case of cells with an expected count <5. Statistical analysis was conducted using the SPSS statistical software for Windows, version 24.0 (SPSS, Chicago, IL, USA) and a value of p<0.05 was considered significant.

This study protocol was approved by the Institutional Review Board (IRB) of Gyeongsang National University Changwon Hospital and the requirement for informed consent waived (GNUCH 2020-07-014). All methods were performed in accordance with the relevant guidelines and regulations of institution. All procedures carried out in this study involving human participants in need of ethical approval were in accordance with the Declaration of Helsinki in 1964 and subsequently amended or similar ethical standards.

## Results

A total of 244 patients were included in this study. Demographic and histopathological characteristics for all patients are shown in Table 1 and Table 2. Group A consisted of 51 (20.9%) patients and group B consisted of 193 (79.1%) patients. In group A, type I and type II endometrial cancers included 41 patients (80.4%) and 10 patients (19.6%), respectively. Group A was significantly older than group B (median age: 61.6 vs 56.6 years, p=0.001). There was no significant difference between group A and group B with regards to parity, weight, BMI and menopausal age. There was a significant association between endometrial cancer and the presence of hypertension (p=0.000). In contrast, the presence of diabetes or thyroid disease was not significantly associated with endometrial cancer (p=0.773 and p=0.537, respectively). Endometrial cancer was significantly related to the history of previous endometrial hyperplasia (p=0.004) in 10 (19.6%) and 11 (5.7%) patients in group A and group B, respectively. The most common symptom in both groups was vaginal bleeding, which was showed by 37 patients (72.5%) and 106 (54.9%) patients in group A and group B, respectively. Other symptoms included abdominal pain and vaginal discharge. There were three patients in group A and 65 patients in group B without specific symptoms.

**Table 1:** demographic and clinical characteristics of 244 postmenopausal patients screened for endometrial cancer from February 2016 to December 2019 at Gyeongsang National University Changwon Hospital in South Korea

Characteristics	Group A (n=51)	Group B (n=193)	p-value
Age (year)	61.6 ± 9.7	56.6 ± 6.3	0.001*
Parity	2.4 ± 1.0	2.3 ± 0.7	0.564*
Weight (Kg)	58.8 ± 8.8	59.8 ± 8.0	0.443*
BMI (Kg/m2)	24.2 ± 3.5	24.1 ± 3.1	0.784*
Menopausal age	50.3 ± 4.0	50.7 ± 2.7	0.436*
Duration of menopause	11.3 ± 11.1	5.9 ± 7.1	0.002*
**Underlying condition**			
Hypertension	27 (52.9)	35 (18.1)	0.000†
Diabetes	6 (11.8)	20 (10.4)	0.773†
Thyroid disease	2 (3.9)	15 (7.8)	0.537ǂ
MHT	0 (0)	10 (5.2)	0.127ǂ
Tamoxifen use	5 (9.8)	8 (4.1)	0.153ǂ
Hx of endometrial hyperplasia	10 (19.6)	11 (5.7)	0.004ǂ
**Operation history**			
Gastric cancer	3 (5.9)	4 (2.1)	0.161ǂ
Breast cancer	7 (13.7)	10 (5.2)	0.057ǂ
Thyroid cancer	0 (0)	8 (4.1)	0.210ǂ
**Symptom**			
Vaginal bleeding	37 (72.5)	106 (54.9)	0.023†
Abdominal pain	4 (7.8)	9 (4.7)	0.480ǂ
Vaginal discharge	7 (13.7)	13 (6.7)	0.146ǂ
No symptom	3 (5.9)	65 (33.7)	0.000†

Group A included patients with endometrial cancer; group B included patients with non-malignant lesions; values are presented as mean ± standard deviation or number (%); *p-value obtained by student t-test; †p-value obtained by Chi-square test; ǂp-value obtained by Fisher's exact test; p-value of less than 0.05 was considered to be significant; MHT: menopause hormonal therapy; Hx: history

**Table 2:** histopathologic characteristics of 244 postmenopausal patients screened for endometrial cancer from February 2016 to December 2019 at Gyeongsang National University Changwon Hospital in South Korea

	Number (%)	Mean value of EM thickness (mm)
Endometrial cancer	51	
Type 1	41	
Endometrioid	30 (58.8)	18.3
Adenocarcinoma	11 (21.6)	17.4
Type 2	10	
Serous	6 (11.8)	18.7
Carcinosarcoma	4 (7.8)	28.0
Non-cancerous lesion	193	
Endometrial polyp	101 (52.3)	13.7
Submucosal myoma	30 (15.6)	23.6
Endometrial hyperplasia	18 (9.3)	21.0
Atrophic endometrium	9 (4.7)	10.9
Other	35 (18.1)	10.9

EM: endometrial thickness

Ultrasound characteristics of both groups are listed in Table 3. The ET was significantly thicker in group A than group B (p=0.028), with a median ET of 18.9 mm (range: 7.5-45 mm) in group A and 15.3 mm (range: 1.6-72.1 mm) in group B. Patients with 10 mm ≤ET <20 mm were the most common in both groups, with 22 patients (43.1%) in group A and 78 (40.4%) in group B. Statistically significant differences in ET were seen in group B with 5 mm ≤ET <10 mm (7 vs 57 patients, p=0.022) and group A with 20 mm ≤ET <30 mm (18 vs 40 patients, p=0.030). There were no statistically significant differences in ET ≤30 mm (4 vs 14, p=1.000) in both group. Group B had significantly more patients with polypoid mass-like lesions (5 vs 96, p=0.000). Endometrial fluid collection showed statistically significant differences in group A and group B with 12 (23.5%) patients and 6 (3.1%) patients, respectively.

**Table 3:** ultrasound characteristics of 244 postmenopausal patients screened for endometrial cancer from February 2016 to December 2019 at Gyeongsang National University Changwon Hospital in South Korea

	Group A (n=15)	Group B (n=193)	p-value
Endometrial thickness (ET)	18.9 ± 8.3	15.3 ± 10.8	0.028*
ET <5mm	0 (0)	5 (2.6)	0.587†
5 ≤ET <10mm	7 (13.7)	57 (29.5)	0.022ǂ
10mm ≤ET <20mm	22 (43.1)	78 (40.4)	0.725ǂ
20mm ≤ET <30mm	18 (35.3)	40 (20.7)	0.030ǂ
ET ≥30mm	4 (7.8)	14 (7.3)	1.000†
Polypoid mass like lesion	5 (9.8)	96 (49.7)	0.000*
Endometrial fluid collection	12 (23.5)	6 (3.1)	0.000†

Group A included patients with endometrial cancer; group B included patients with non-malignant lesions; values are presented as mean ± standard deviation or number (%); *p-value obtained by student t-test; †p-value obtained by Fisher's exact test; ǂp-value obtained by Chi-square test; p-value of less than 0.05 was considered to be significant

## Discussion

Endometrial cancer occurs mainly in the postmenopausal period and more than 90% of women are diagnosed at an age >50 years, with a mean age of 63 years [2]. In this study, the median age at the time of diagnosis was 61.6 years (range: 50-83 years). The mean age of group A was higher than that of group B (61.6 vs 56.6). This is consistent with the fact that the incidence of endometrial cancer increases with age. In group A, median age of type 1 and type 2 were 60.9 years and 63.4 years, respectively. Risk factors such as obesity, diabetes, thyroid disease, tamoxifen use or late menopause are known to be associated with endometrial cancer, but were not relevant in this study. These results were probably because patients in group B included those with endometrial polyps or endometrial hyperplasia associated with unopposed exposure of estrogen. In addition, patients with type II endometrial cancer, which usually only accounts for about 10% of endometrial cancers and are known to be unrelated to the unopposed exposure of the endometrium to estrogen, comprised a high percentage (19.6%) in this study. Risk factors such as hypertension or a previous history of diagnosed endometrial hyperplasia were associated with endometrial cancer in this study. Hypertension was considered an independent risk factor for endometrial cancer [13], but some studies claimed that hypertension was a risk factor for endometrial cancer due to accompanying diabetes or obesity [2]. In our study, BMI in patients with hypertension was 24.24 kg/m^2^ in group A, 23.27 kg/m^2^ in only type 1 patients excluding type 2 and 25.66 kg/m^2^ in group B. This fact shows that patients with hypertension in group A are not obese when compared to those in group B. Among hypertensive patients, 4 (4/27, 14.8%) patients in group A and 22 (22/35, 62.9%) patients in group B were accompanied with diabetes. Hypertension appeared to be an independent factor in development of endometrial cancer in this study. Another risk factor is a previous diagnosed history of endometrial hyperplasia. However, since this study was conducted through previous medical records, the type of hyperplasia and interval to the current histologic results were not obtained.

Vaginal bleeding is a common symptom of endometrial lesions in postmenopausal women. Postmenopausal vaginal bleeding (PMB) is associated with benign conditions such as atrophic endometrium or endometrial polyp, but 3-25% of these patients are diagnosed with endometrial cancer [14-17]. A total of 143 patients presented with vaginal bleeding in this study. Among these patients, 37 (25.8%) were diagnosed with endometrial cancer. The prevalence of PMB in patients with endometrial cancer has been reported to be 90%, which showed no significant differences based on cancer stage [18]. Seven patients (21.5%) in group A had nonspecific symptoms such as abdominal pain (n=4) or no symptoms (n=3), which was not related to a gynecological problem. Among the asymptomatic patients, three patients were diagnosed with endometrial cancer. Histological types of all three cases were endometrioid adenocarcinoma. These patients had ET measuring 20 mm, 20 mm and 33.8 mm. Among the asymptomatic women in both groups, the presence of endometrial polyps was the most common symptom (n=35 patients). Patients diagnosed with endometrial cancer show various symptoms as well as vaginal bleeding. It doesn't seem right to decide whether to perform a test to diagnose endometrial cancer based on symptoms such as vaginal bleeding. ET measured by TVUS should be recommend as a screening test for endometrial cancer in asymptomatic postmenopausal women. There has been controversy regarding the cutoff value of ET, which is a required invasive test for endometrial pathology. The American College of Obstetricians and Gynecologists recommends that patients with PMB should be tested for endometrial cancer only in cases with ET >4 mm [9], but other studies have reported that 96% of patients diagnosed with endometrial cancer showed an ET >5 mm [10]. In another recent meta-analysis, the sensitivity of diagnosis of endometrial cancer was 90% when the cutoff value of ET was >5 mm, but the sensitivity was 97.9% (95% confidence interval: 90.1-99.6%) when the cutoff value of ET >3 mm. The authors, therefore, suggested an ET cutoff value of >3 mm [11]. In the present study, two patients with ET <3 mm, five patients with ET <4 mm and eight patients with ET ≤5 mm underwent histological clarification, with no diagnosis of endometrial cancer. Among the patients diagnosed with endometrial cancer, the minimum ET was 6 mm and seven patients were diagnosed when the ET was 5 mm ≤ET <10 mm; therefore, our results suggested an ET cutoff value ≥5 mm for further invasive procedures to diagnose endometrial cancer. In patients with PMB, endometrial cancer increases in proportion to the increase of ET, especially when the ET was >20 mm (19-21). Among patients with vaginal bleeding in this study, 40 patients had an ET ≥20 mm and 103 patients had an ET <20 mm. Fourteen (35%) patients in the former case and 23 (22.3%) patients in the latter case were diagnosed with endometrial cancer.

Among postmenopausal women, uterine fluid collection during ultrasound examination was observed in 7.8% of the patients [19]. EFC may result from several causes such as cervical stenosis, hematometra, pyometra and endometrial malignancy [20-22]. There are equivocal data concerning the clinical significance of EFC in postmenopausal women and whether further invasive methods for endometrial histological evaluation should be performed. In one study on EFC in an elderly population, only one out of 31 women was diagnosed with endometrial cancer; therefore, the authors concluded that the ET was more important than EFC in endometrial abnormalities [23]. In another study, EFC did not increase with the risk of malignancy when the ET was thin in asymptomatic postmenopausal women [24]. Some authors have reported that further examination was not necessary if the ET was <3 mm. They suggested that the ET or general characteristics of the surrounding tissue was more important than the presence of EFC [25]. For postmenopausal women with EFC, histological evaluation such as endometrial sampling is recommended only when the endometrial thickness is >3 mm and the endometrial fluid is echogenic [26]. In a study of 52 asymptomatic postmenopausal women with EFC, no patient was diagnosed with endometrial cancer.

An endometrial histopathological examination was not required for patients with an ET <3 mm [27]. However, some studies have recommended that endometrial sampling should be performed in all cases with EFC regardless of ET, because the EFC can mask endometrial pathology due to the pressure effect on the endometrial lining [28]. Of the 18 patients with EFC in our study, there were 12 (66.6%) patients in group A and six (33.4%) patients in group B. The ET of all patients was more than 6 mm. All patients in group B were diagnosed with endometrial polyp. This result shows that EFC with increase of ET was a risk factor of endometrial cancer. When patients with an increase of ET show EFC, further invasive methods should be recommended to identify endometrial malignancy. Among patients with polypoid mass-like lesions, which was the other ultrasound characteristic that was analyzed, there were five (9.8%) patients and 96 (49.7%) patients in group A and group B, respectively. Patients with no malignant lesions were found in significantly higher than patients with malignancy (p=0.001). This fact shows that polypoid mass-like lesions, focal thickening of the endometrial layer, were not risk factors of endometrial malignancy.

## Conclusion

The present study showed that older age, hypertension and a previous diagnosed history of endometrial hyperplasia are risk factors for endometrial cancer. We could not find any significant association between endometrial cancer and the well-known risk factors such as obesity, diabetes and tamoxifen use. In postmenopausal women, endometrial cancer was more associated with vaginal bleeding when compared with benign endometrial lesions. Because asymptomatic patients were also diagnosed with endometrial cancer, we recommend more use of TVUS as a preoperative diagnostic tool for endometrial cancer. No endometrial cancer was found in patients with an ET <5 mm, but endometrial cancer was diagnosed in all ranges when the ET was ≥5 mm. In addition, there was a greater association with endometrial cancer in the presence of EFC, in cases with increased ET. In patients with an ET ≥5 mm detected, further invasive procedures are recommended to rule out the possibility of an endometrial malignancy. Regardless of symptoms and risk factors, ET in postmenopausal women should be measured by TVUS to screen for endometrial cancer; if a thickened ET and other abnormalities are found, tests for endometrial histological confirmation should be conducted immediately.

### What is known about this topic

- Endometrial cancer is known to be associated with risk factors such as obesity, diabetes mellitus, tamoxifen use or late menopause;
- There are several cutoff values of endometrial thickness measured by transvaginal ultrasound that recommend histological examination for the diagnosis of endometrial cancer.

### What this study adds

- Hypertension is an independent risk factor of endometrial cancer;
- When endometrial thickness is increased, the fluid collection in endometrial cavity seen on ultrasound is more likely to be malignant lesions rather than benign lesion.
